# Lessons from SARS-CoV-2 Pandemic: Evolution, Disease Dynamics and Future

**DOI:** 10.3390/biology9060141

**Published:** 2020-06-26

**Authors:** Saurabh Pandey, Bharat Yadav, Arvind Pandey, Takshashila Tripathi, Masuma Khawary, Sashi Kant, Deeksha Tripathi

**Affiliations:** 1 Department of Biochemistry, School of Chemical and Life Sciences, Jamia Hamdard, New Delhi-110062, India; saurabhpandey@jamiahamdard.ac.in; 2 Microbial Pathogenesis and Microbiome Lab, Department of Microbiology, Central University of Rajasthan, Ajmer, Rajasthan-305817, India; yadavbharat22@gmail.com (B.Y.); 2019phdmb05@curaj.ac.in (M.K.); 3 Department of Statistics, Central University of Rajasthan, Ajmer, Rajasthan-305817, India; arvindpandey@curaj.ac.in; 4 Department of Neuroscience, Physiology and Pharmacology, University College London, London WC1E 6BT, UK; t.tripathi@ucl.ac.uk; 5 Department of Immunology and Microbiology, University of Colorado School of Medicine, Anschutz Medical Campus, Aurora, CO 80045, USA

**Keywords:** COVID-19, SARS-CoV-2, pandemic

## Abstract

The COVID-19 pandemic is rising at an unprecedented rate. The surging number of deaths every day, global lockdown and travel restrictions have resulted in huge losses to society. The impact is massive and will leave a historical footprint. The Spanish Flu of 1918, which was the last pandemic that had a similar impact, was shadowed under the consequences of World War I. All the brilliance, strength and economies of countries worldwide are aimed at fighting the COVID-19 pandemic. The knowledge about coronavirus dynamics, its nature and epidemiology are expanding every day. The present review aims to summarize the structure, epidemiology, symptoms, statistical status of the disease status, intervention strategies and deliberates the lessons learnt during the pandemic. The intervention approaches, antiviral drug repurposing and vaccine trials are intensified now. Statistical interpretations of disease dynamics and their projections may help the decision-makers.

## 1. SARS-CoV-2, Structure, Epidemiology and Symptoms of COVID-19

### 1.1. SARS-CoV-2: Structure and Similarity to Other Coronaviruses

The infection caused by novel coronavirus, SARS-CoV-2, COVID-19 has turned into a pandemic. SARS-CoV-2 was isolated from the samples of 4 patients in Wuhan on 21 December 2019 [1]. SARS-CoV-2 is an enveloped, positive, single-stranded RNA virus [2,3]. It appears that SARS-CoV-2 is an animal coronavirus that originated from a bat and phylogenetically belongs to the genus beta-coronavirus (subgenus: Sarbecovirus) and shares considerable similarities to human coronaviruses responsible for earlier outbreak: SARS and MERS [1,4]. SARS-CoV-2 shares 96.3%, 89%, and 82% nucleotide similarity with bat CoV RaTG13, SARS-like CoV ZXC21, and SARS-CoV, respectively [5]. Though SARS-CoV-2 is closer to SARS with 77.2% amino acid similarity [4]. The spike proteins of both are homologous with 80% identity and are responsible for binding to the receptor, and membrane fusion. SARS-CoV-2 use angiotensin-converting enzyme 2 (ACE2) mammalian receptor like SARS-CoV for cellular entry for human-to-human transmission [3,4,6]. However, SARS-CoV-2 possesses a longer spike protein in comparison to SARS-CoV [3]. Being lineage B (subgenus Sarbecovirus) of betacoronaviruses, that includes SARS-CoV-2 and Bat SL-CoV-WIV1, their primary mode of transmission is through droplet and fecal-oral route [7]. SARS-CoV-2 infects the lung alveolar epithelial cells using receptor-mediated endocytosis via angiotensin-converting enzyme II (ACE2) as an entry receptor [2]. Further, the higher rate of infectivity of SARS-CoV-2 has been attributed to higher rigidity of the outer protein coat which makes it more resilient as compared to SARS and MERS [8]. The higher infectibility of SARS-CoV-2 could also be due to its lower fatality rate as compared to SARS and MERS [9]. SARS affected 29 countries whereas MERS affected 27 countries and were responsible for 774 and 858 deaths, respectively, around the globe [10]. The latest situation reports by World Health Organization (WHO) on the COVID-19 pandemic states that it has already claimed more than 353,334 lives worldwide which is manifold greater than the total amount of fatalities of SARS and MERS combined [3,11]. The initial mode of virus entry from bats to humans at Huanan Seafood Market is still not clear [3]. It is expected that an intermediate organism might be involved in this transmission.

The mutation rates in RNA viruses are as high as one million-fold greater than their host. Mutation-selection forces keep the virus close to threshold beyond which they may go extinct due to large scale deleterious mutations [12]. In the case of SARS-CoV-2, mutation rates are high, including spike glycoprotein that is the ACE2 binding region important for virus entry into the cells. Other highly mutated regions are ORF1ab, ORF8 and NSP-1 alongside the spike glycoprotein region [13]. These overwhelming mutations are the leading cause of concern in the process of development of therapeutic interventions. Also, any gain of function mutation at vital cellular entry pathways of SARS-CoV-2 can make the virus more infectious. Furthermore, the combination of antivirals administered to COVID-19 patients may enhance the mutation rate [14,15]. The prevalence and genetic diversity of SARS-CoVs in humans, bats and other mammals and their proximal existence will cause frequent recombination and spillovers and will become the source of future infections as noted by Dr. Zheng-Li while describing the genetic evolution of coronaviruses [16].

### 1.2. Epidemiology

SARS-CoV-2 and previous outbreaks of coronaviruses are suspected to be originated from bats due to frequent recombination and spillover of coronavirus causing COVID-19 in humans [16]. After possible recombination events, it might have jumped to new species via intermediate host like civets (*Paguma larvata*) or pangolin and then reached humans at a seafood market in Wuhan, China [17,18,19,20]. Though, it cannot be said with full convection that civet cat or pangolin are the true intermediate hosts. The ability to survive in human hosts has instigated the explosive spread of SARS-CoV-2. It can infect anyone of us, but comorbidities like hypertension, diabetes, respiratory symptoms and kidney disease are significant risk factors and may result in severe clinical manifestation [21,22].

The earliest cases of COVID-19 were reported from China in December 2019 [1]. WHO failed to recognize the condition that eventually grew to the pandemic scale [10,23,24]. Initial opposition to imposing travel restrictions and negating human to human transmission of SARS-CoV-2 are examples of this [24]. WHO declared COVID-19 a Public Health Emergency of International Concern on 30 January 2020. However, it was not before 11 March 2020, that WHO declared it a pandemic [2,10,11].

According to the WHO situation report on COVID-19 dated 28 May 2020, a total of 5,593,631 confirmed cases with 353,334 deaths have been reported globally. The USA alone has reported 1,658,896 confirmed cases and 98,119 deaths due to COVID-19 so far [11]. The majority of cases have been reported from European and American continents, whereas numbers are less in African and Asian continents but are increasing rapidly. Globally, cases are doubling every 4–5 days [11,25]. African and Asian nations are at higher risk due to their large population and poor healthcare facilities. COVID-19 has already surpassed SARS and MERS in quantity of cases and deaths, although it has a lower fatality rate than the latter two [3,10,11]. SARS-CoV-2 has spread at a much higher pace compared to previous human bat coronaviruses [8]. The higher infectibility of SARS-CoV-2 can be attributed to its ability to remain infectious outside the human body for a long duration of time. Van Doremalen et al. demonstrated that under similar incubation conditions of temperature and humidity, over 7 days, SARS-CoV-2 remain viable on common surfaces such as plastic and stainless steel for 6.8 and 5.6 h, respectively [8,26]. It has a 14 day long incubation period which can extend up to 27 days, increasing the chances of its spread due to asymptomatic carriers of the disease present in a population [5,9,11,27,28,29,30]. A Chinese research group has reported presence of SARS-CoV-2 in the stool of patients. This can amount to a greater severity of infectivity of the virus, as the chance of spread through urban sewage is always there [9].

Lockdown employed by China to mitigate the disease has shown positive results in decreasing the growth rate and increasing the doubling time of COVID-19 in China [31]. Although everyone is equally susceptible to SARS-CoV-2, the majority of the cases lie in the age group 30–79 years, as shown in 87% of cases in the Chinese Center for Disease Control and Prevention Report referred by Zunyou Wu, and also infections in males are higher [9,10,32]. SARS-CoV-2 may also affect neonates but it is unclear if the transmission is vertical, from mother to child, or by infected individuals [33].

While the world is still struggling to fight this pandemic, several cases of recurrence of SARS-CoV-2 in recovered patients have emerged as another challenge. In Guangdong, China, 20 out of 182 patients who had completely recovered from the infection and were discharged from hospital were found to be positive when re-tested. They didn’t show any clinical symptoms. A notable case from this group is of an 8-year-old boy who consistently re-tested positive even 35 days after his first discharge from hospital [34]. 

A Malayan tiger, Nadia at the Bronx Zoo, New York tested positive for SARS-CoV-2 [35]. A research group at the Harbin Veterinary Research Institute, China reported that cats and ferrets were susceptible to SARS-CoV-2 infection whereas dogs, pigs, chickens and ducks were not permissive to infection [36]. The World Organization for Animal Health has reported SARS-CoV-2 infection in farmed minks, dogs, golden Syrian hamsters, cynomolgus and rhesus macaques. Although there are no significant reports about their epidemiological role in the spread of the disease among humans [37], these developments now raise questions over infective capacity of the virus and the probable role of animals in the spread of the virus.

Diagnosis of SARS-CoV-2 positive cases is instrumental in fighting COVID-19. Though RT-PCR based molecular diagnostic tests remain a gold standard for diagnosis of this virus, new serological tests which detect IgM and IgG antibodies in the patient sample formed against nucleocapsid protein or spike protein of the virus are also in use [38].

The coronavirus SARS-CoV-2 has affected more than 210 countries and territories around the world. Up until 28th May 2020, a whopping 5,593,631 corona infection cases are reported. Among them, 95% of cases are from 45 countries. Only sixteen countries (USA, Brazil, Russia, Spain, UK, Italy, France, Germany, India, Turkey, Iran, Peru, Chile, Canada, China and Saudi Arabia) are contributing to 80% of the total number of cases in the world.

**Figure 1:**
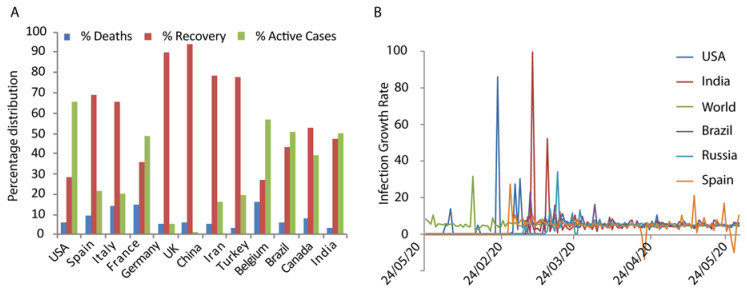
(**A**) Percentage distribution of number of deaths, recovery and active cases in worst-hit countries in the world. (**B**) Infection growth rate of some of the worst infected countries is shown. The data represent quite a linear growth.

Around the globe, the number of reported deaths is 353,334 till 28 May 2020. Also, the contribution of the previously mentioned countries (USA, Brazil, Russia, Spain, UK, Italy, France, Germany, India, Turkey, Iran and Peru) to total deaths due to corona infection is nearly 80%. Only seven of the above countries (USA, Brazil, Russia, Spain, UK, Italy and France) are contributing to more than 72% of the overall deaths in the world due to corona infection [39]. The distribution of deaths, recovery and active cases in highly affected countries in the world is shown in Figure 1A.

It is observed that males and females are equally sensitive to get infected by coronavirus [40]. Men are slightly more prone to get infected. But when it comes to the number of deaths, there is a significant difference between the genders. Although, the reason for such significant difference in the death rate of different gender is not known. The rate of infection in different countries per 100,000 is similar on the basis of age and sex. Germany does not have much variation based on sex and age in the number of cases. But for the age group greater than 60, the number of deaths per 100,000 population is very high. On the other hand, in Italy, for the age group greater than 50, the number of infection and death per 100,000 population is very high. In China, the ratio of male and female death in confirmed cases is 17:10. In India, the working population is more affected with those aged 21 years to 60 years contributing to 75% of the total number of infected cases [41]. The daily growth rate excluding China reflects that the rate of increase is quite stable; it is not as alarming as at the beginning (Figure 1B).

### 1.3. Symptoms

COVID-19, SARS and MERS share a number of common symptoms, fever, dry cough, dyspnoea and bilateral ground-glass opacities in the lungs [42]. Discomfort in breathing is also reported among the patients. Less common symptoms are headache, fatigue, chest tightness and diarrhea [9,24]. Previously, diarrhea was reported in patients suffering from MERS but not SARS [9]. It was reported from blood examination of the patients that they also had leucopenia, mild lymphopenia, elevated levels of aspartate aminotransferase (AST), lactic dehydrogenase (LDH), γ-glutamyl transpeptidase (γ-GT) and α-hydroxybutyric dehydrogenase (α-HBDH) [4,24,42]. In acute cases, it could lead to pneumonia, liver damage, kidney failure and death [42,43]. Acute liver damage in patients with SARS or Influenza has been reported in the past [24]. Severe cases of COVID-19 yield multiple organ dysfunction syndrome (MODS), acute respiratory distress syndrome (ARDS), septic shock, acidosis and cytokine storms in the body which may lead to death [44,45]. COVID-19 patients also exhibited bilateral focal consolidation, ground-glass opacities, lobar consolidation and diffused patchy consolidation in chest radiography [4]. The three viruses have similar zoonotic transmission from mammals to humans but unlike SARS and MERS, SARS-CoV-2 rarely causes runny nose and gastrointestinal symptoms [43].

Several asymptomatic individuals were also tested positive for SARS-CoV-2. In a comprehensive study by Chinese Center for Disease Control and Prevention (China CDC) involving 72,314 SARS-CoV-2 positive cases, it was observed that 1.2% (889) individuals were asymptomatic and showed no clinical symptoms [10]. Also, 30.8% of 565 Japanese citizens evacuated out of Wuhan, China were reported to be asymptomatic carriers of the disease [46]. The total number of such asymptomatic cases pan globe is unknown at this point of time. The asymptomatic carriers pose a huge challenge to the containment of this pandemic.

## 2. Status of Human Interventions

### 2.1. Drugs and Treatment

There is no specific drug or vaccine available for SARS-CoV-2 for use in humans. The present treatment employs two-pronged approach—use of antibiotics to prevent secondary infections and use of available antiviral drugs to eliminate the virus. Although, no antiviral drug has presented conclusive results with sufficient scientific data as a treatment against COVID-19 [43,47]. Alternatively, repurposing of available antiviral drugs is under trial. WHO has launched SOLIDARITY trials, an internationally coordinated trial to find effective drugs against SARS-CoV-2. It relies on the repurposing of antiviral drugs already approved for use in humans [26]. The drugs included in the SOLIDARITY trial are lopinavir and ritonavirplus interferon beta as well as chloroquine, and remdesivir. Lopinavir and ritonavir, remdesivir inhibit viral replication. Patients have shown positive responses to treatments with a combination of lopinavir and ritonavir [2]. Remdesivir has shown high efficacy against other coronaviruses which makes it a strong candidate for use against SARS-CoV-2 [23]. Monoclonal antibodies such as Tocilizumab can be useful to counter cytokine storms in severe patients [26]. Presently, a number of clinical trials registered on ClinicalTrials.gov include immunoglobulins, remdesivir, arbidol hydrochloride combined with interferon atomisation, hydroxychloroquine, ritonavir plus oseltamivir and many more [2]. Hydroxychloroquine, an anti-malarial and anti-inflammatory drug inhibits viral replication by increasing the pH of endosomes that house the virus inside the cell and has shown promising results against SARS-CoV-2 in in-vitro studies. Since then, its demand has increased globally [26]. A recent study conducted upon a group of 80 individuals showed improvement in symptoms upon administration of hydroxychloroquine and azithromycin [48]. However, there are still no convincing studies to establish hydroxychloroquine as the therapeutic candidate for SARS-CoV-2 and further investigations are required involving larger groups. The Indian Council of Medical Research (ICMR) has approved the clinical trial of live attenuated Mycobacterium W (*Mycobacterium indicus pranii*), commercially available as Sepsivac (Cadila Pharmaceuticals) to be tested on COVID-19 patients, their close contacts and healthcare workers. This has been used previously for leprosy, advanced non-small cell lung cancer and severe gram-negative sepsis.

Presently, several research groups are engaged in the development of a non-human primate model to study SARS-CoV-2 infection for testing potential vaccines and antivirals. In addition to providing a better understanding of virus–host interactions, it will prove instrumental in drug and vaccine development [43]. Macaques and African green monkeys have identical sequences of cell surface proteins that may allow the entry of SARS-CoV-2. African Green, Rhesus and Cynomolgus Monkeys have been previously tested for successful replication of SARS virus [49]. After infection, the maximum amount of serum neutralizing antibody is produced in African green monkey, followed by cynomolgus and rhesus macaques against SARS infection, respectively [49]. Though the infection model for SARS-CoV-2 in these non-human primates shows successful replication, the African green monkey appears as a better model as it requires lower, close to natural dose for infection and develops advanced respiratory disease [50]. Variation in level of viral replication and subsequent clinical manifestation may be a challenge for effective use of these models.

### 2.2. Vaccine Trials and Challenges

In the absence of any effective antiviral treatment for SARS-CoV-2, development of an effective vaccine seems imperative to tackle the pandemic situation. Presently, there are no vaccines available for COVID-19 in the world. Spike protein plays a major role in receptor recognition, viral invasion into the cell and is also exposed to immune reactions. Therefore, it seems to be a suitable target for vaccine development. Similarity in the spike proteins of SARS, MERS and SARS-CoV-2 will help scientists to repurpose or utilize the experience from the previous two viruses to develop a vaccine for COVID-19 [3,6,26]. The scientific community worldwide has accelerated efforts to develop a vaccine against COVID-19 using all possible strategies—live/attenuated virus vaccine, subunit vaccine and nucleic acid vaccines [51].

**Figure 2:**
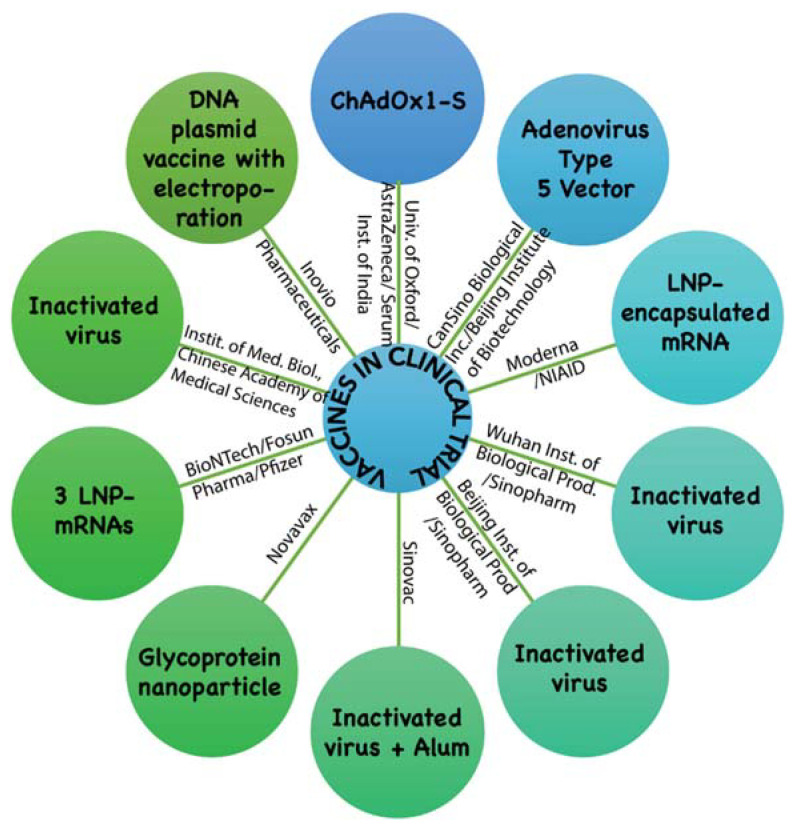
Ten vaccine candidates are in clinical trial, as shown in the figure. Information was accessed and adapted from DRAFT landscape of COVID-19 candidate vaccines—prepared by WHO, accessed on 27 May 2020 [52].

As per the WHO update, dated 27 May 2020, ten vaccine candidates have entered clinical trials in different countries (Figure 2). In the USA, trials have begun for an RNA vaccine, mRNA-1273 developed by the US National Institute of Allergy and Infectious Diseases (NIAID) in partnership with Moderna. An adenovirus based vaccine named Ad5-nCoV developed by CanSino Biologics (Tianjin, China), has entered clinical trials in Wuhan, China and a DNA based vaccine developed by Inovio Pharmaceuticals has entered Phase I clinical trials in South Korea [52]. Indian company Serum India Limited is also participating in a ChAdOx1-S vaccine development program. Along with 10 clinical trial candidate vaccines, another 115 candidate vaccines are in the pre-clinical stage of development as per DRAFT landscape of COVID-19 candidate vaccines—prepared by WHO, accessed on 27 May 2020 [52] (Table 1).

Many pharmaceutical firms have come forward to develop a vaccine against COVID-19. CureVac, Tübingen, Germany and Inovio Pharmaceuticals Inc., Plymouth Meeting, Pennsylvania, USA/Beijing Advaccine Biotechnology Co., Beijing, China with funding from the Coalition for Epidemic Preparedness Innovations (CEPI) is working on separate nucleic acid vaccines [48]. Janssen (Johnson & Johnson), Beerse, Belgium and Codagenix Inc., Farmingdale NY, USA/Serum Institute of India, Pune, India in collaboration with the University of Queensland, are working towards developing whole virus vaccines, whereas CEPI, Davos, Switzerland; Novavax, MD, USA; Clover Biopharmaceuticals, Chengdu, China; Vaxart Inc, San Francisco, CA, United States and Sanofi Pharmaceuticals, Paris, France are in efforts to develop vaccines using recombinant proteins for COVID-19 [26,52].

Development of a vaccine against coronaviruses may face challenges like antibody-dependent enhancement. In antibody-dependent enhancement, the antibody itself facilitates the viral entry into the cells via Ig Fc receptors [53,54]. The same has been observed in the case of veterinary vaccines against animal coronaviruses, including feline infectious peritonitis virus [54]. Further, studies of animal coronaviruses have established pathogenicity, cell-tropism genotype diversity and evolution for animal coronaviruses in the last two decades. Evolutionary forces of mutation and recombination on generating new strains and diversity of strain types in the same geographical location and availability of same strains at faraway distances increase the opportunity of a SARS-CoV-2 like outbreak [16,55]. Even though several vaccines are under trial and many are under development (Figure 2), they may not be available for the public before 2021 [23]. Therefore, deterrence of human-to-human contact, decreasing mortality and successful repurposing of available drugs remains vital in fighting this pandemic.

## 3. Lessons from SARS-CoV-2 Pandemic

**Figure 3:**
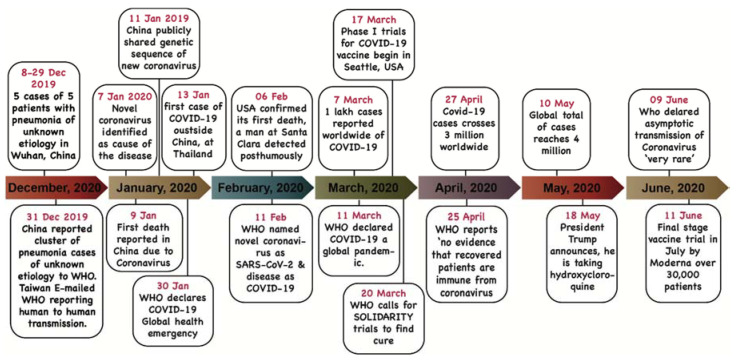
The timeline represents the important events during the novel coronavirus born pandemic till June 2020.

SARS-CoV-2 has surpassed previous coronavirus outbreaks of the past in terms of total deaths, number of affected individuals; though it has lower CFR (case fatality rate) [3,10,24]. Restricted and inadequate information from the epicenter of the outbreak affected the early warning and preparedness and resulted in the burst of COVID-19 cases globally. Important events of COVID-19 pandemic in last few months are shown in the form of a timeline (Figure 3). However, by the time COVID-19 was declared a pandemic, it had already spread to many countries, owing to extensive international connectivity of China to the world via international trade and tourism [23,24].

In the past, travel restrictions have shown a positive effect in containing previous epidemics of SARS, Ebola and plague. Hence, immediate travel restrictions should have been imposed but were delayed and not put in place by the country of initial outbreak [32]. Even when they declared the national medical emergency, the international community failed to suspend trade and travel activities [23]. Greed for monetary affairs and domination has worsened the situation by carrying the virus out pan globe.

European nations didn’t impose a complete lockdown, hence despite having advanced healthcare services, they have suffered huge losses of life [11,24]. The present pandemic has reminded us about the need for quick response mechanisms both at international and national levels to mitigate future outbreaks. There is a pressing need for coordinated research and dedicated funds to counter the pandemic and similar situations in the future. Strengthening the research and development sector along with the medical healthcare system will be the next step of the post-pandemic era.

The development of fast, reliable and cost-effective diagnostic methods is a matter of priority. To find a cure for COVID-19, vaccine development and repurposing of drugs as antivirals against SARS-CoV-2 have been started, but a major hurdle is the unavailability of a suitable animal model to study the disease and pre-clinical trials [43]. In absence of any reliable treatment, identification in infected individuals and their subsequent isolation is the best bet to contain the pandemic. Current diagnostic approaches for detection of SARS-CoV-2 infections are based on RT-PCR that detects the presence of viral nucleic acid in the patient sample or a serological approach which detects IgG or/and IgM antibodies formed against the causative viral antigens. Another serological approach relies on detecting the SARS-CoV-2 antigens but has not been utilized much for development of diagnostic kits/methods. Though RT-PCR based diagnosis of SARS-CoV-2 remains the benchmark for diagnosis of the disease, faster serology based diagnostic tests are being used to screen SARS-CoV-2 positive individuals, owing to its ability to give a result in a shorter time compared to RT-PCR based methods, and also its ease of implementation in mass testing for SARS-CoV-2. The diagnostic kits approved by the FDA, USA are based on detecting IgG and IgM antibodies produced in the body in response to SARS-CoV-2 infection. The registered serological kits claim to provide a result in 15 min in the laboratory.

The sub-Saharan nations stand as most vulnerable to the COVID-19 outbreak due to their fragile healthcare system. Therefore, weaker nations stand at high risk and deserve financial and healthcare support from the rest of the world [2]. Developing and underdeveloped nations are facing severe shortage of healthcare facilities, trained workforce and critical medical equipment such as ventilators and diagnostic kits. As per the Forum for Innovation and Diagnostics (FIND), the number of tests performed per 1 million population is very low in such nations.

It can be concluded that among all other factors, delayed response from responsible agencies, putting economic and geopolitical benefits above human life and ill-awareness among the agencies as well as the general public has helped shape the COVID-19 epidemic into a pandemic. Further, many questions remained unanswered at this juncture. Firstly, will the SARS-CoV-2 remain in the population, resurrect time and again to haunt us or disappear? Secondly, is the virus jumping to the host a rare and isolated event or may it occur repeatedly? Also, is the recurrent infection, reinfection or second attack of SARS-CoV-2 possible or not and with what frequency? 

Dr. Chang, in his review published in the New England Journal of Medicine, discussed the reinfections of viruses at length [56]. As he discussed, the reason for reinfection lies in partial/incomplete immunity generated after primary infection. Respiratory track viral infections invade primarily superficial respiratory epithelial cells. Probably, cells containing acquired resistance are replaced by new sensitive cells. Also, serum antibodies with capacity to neutralize virus particles can’t easily access the superficial respiratory epithelial cells. Still locally induced and secreted IgA can directly interact with invading viruses. The status of reinfection in the case of COVID-19 is still debatable. No published data yet satisfy our enquiry with confidence. Though, it is noteworthy to cite the recent non-peer-reviewed preprint (https://doi.org/10.1101/2020.03.13.990226). This suggests reinfection failed when equal doses of SARS-CoV-2 titer were administered in the rhesus macaques study model and they remain asymptotic. Any appearance of a positive test report of discharge patients may be due to false-negative detection of residual RNA fragments of dead virus fragments or patients may not be fully recovered. But, a complete picture is still awaited.

Lastly, there will be no miracles, humanity should learn from this hardship. Responsibility must be settled on those who failed to contain the outbreak, let it grow on as pandemic for economic and geopolitical margins. We expect a great boost in healthcare, hygiene and research in life sciences that will directly change the standard of health status of people in the post-COVID-19 pandemic era.

## Figures and Tables

**Table 1:** Details of vaccines under clinical trial.

Technical Platform	Candidate Vaccine in Clinical Trial	Developer	Current Stage
Non Replicating Viral Vector	ChAdOx1-S	University of Oxford/AstraZeneca, Cambridge, U.K. / Serum Institute of India	Phase2b/3 2020-001228-32 Phase 1/2 2020-001072-15
Non Replicating Viral Vector	Adenovirus Type 5 Vector	CanSino Biological Inc., China/Beijing Institute of Biotechnology, China	Phase 2 ChiCTR2000031781 Phase 1 ChiCTR2000030906
RNA	Lipid nanoparticles (LNP) encapsulated mRNA	Moderna Inc., USA/ National Institute of Allergy and Infectious Diseases (NIAID), USA	Phase 2 (IND submitted) Phase 1 NCT04283461
Inactivated	Inactivated	Wuhan Institute of Biological Products, China/Sinopharm Group Co., Ltd., China	Phase 1/2 ChiCTR2000031809
Inactivated	Inactivated	Beijing Institute of Biological Products/ Sinopharm Group Co., Ltd., China	Phase 1/2 ChiCTR2000032459
Inactivated	Inactivated + Alum	Sinovac Biotech Ltd., China	Phase 1/2 NCT04383574 NCT04352608
Protein Subunit	Full length recombinant SARS-CoV-2 glycoprotein nanoparticle vaccine adjuvanted with Matrix M	Novavax Inc., Maryland, USA	Phase 1/2 NCT04368988
RNA	3 LNP-mRNAs	BioNTech, Germany/Shanghai Fosun Pharmaceutical Co. Ltd., China /Pfizer, USA	Phase 1/2 2020-001038-36 NCT04368728
Inactivated	Inactivated	Institute of Medical Biology, Chinese Academy of Medical Sciences	Phase 1
DNA	DNA plasmid vaccine with electroporation	Inovio Pharmaceuticals Inc., USA	Phase 1 NCT04336410

Note: 115 candidate vaccines are in preclinical evaluation. Information taken and adapted from DRAFT landscape of COVID-19 candidate vaccines—prepared by WHO [52].
